# Medical News and Items

**Published:** 1876-03

**Authors:** 


### Medical News and Items.

On the evening of February 14, the Chicago Society of Physicians and Surgeons had an opportunity of inspecting and carefully examining the person of Catherine Hohmann, who has furnished both spermatic fluid and menstrual blood in the presence of experts.

This monster presents an example of bisexual hermaphrodism which is of such rarity that but two, or at the most three, similar cases have ever been recorded.

The readers of the JOURNAL AND EXAMINER, for 1875, will recall a description of this case in Obs. V. of the article on "Hermaphrodism from a Medico-Legal Point of View," translated from the French by Edw. Warren Sawyer, M.D. The original was obtained from a description by Ceccherelli in *Lo Sperimentale.*

---

WE take pleasure in announcing daily additions to our list of foreign exchanges. Among those of the last month are the time-honored *Edinburgh Medical Journal; Giornale della R. Accademia di Medicina di Torino; Anales de Ciencias Médicas; Anales de la Asociacion Larrey; Gazetta Medica Italiana, Lombardia; Archives Génerales de Médecine; Gazette Obstétricale et Gynécologique; Annales de Dermatologie et de Syphiligraphie; L' Union Médicale; Lyon Médical;* and the *Journal de Médecine et de Chirurgie.*

---

AN AUSTRIAN PHARMACEUTIST, in Vienna, has adopted the plan of indicating on the labels of jars and bottles the weight of the *maximum* pharmacopœial doses. The dispenser is thus warned at once if he meet with a prescription in which the maximum dose is exceeded, and can institute measures to assure himself against fatal errors by the writer.

THE EXERCISES of the Twenty-Third Annual Commencement of Rush Medical College, were held on Tuesday Evening, Feb. 15, 1876, in Martine's Hall, cor. 22nd street and Indiana avenue.

Professor F. W. Fiske made the opening prayer, and was followed by President Freer, who delivered an appropriate address. Prof. J. Adams Allen then succeeded, with an address in which he sketched the history of the College and aroused the mirth of his auditors by the fascinations of that sparkling humor which is so characteristic of the speaker. The President then conferred the degrees, and was followed by Prof. E.L. Holmes, who made the third address of the evening, after which the benediction was pronounced. The literary portions of the programme were interspersed with music.

The occasion was one of the happiest that has occurred since the destruction of the old building by fire, and will be long remembered with pleasure by all who were present.

The following are the names of the graduating class:

WELLS ANDREWS, JR.
BENSON BANTON.
IRA BISHOP.
DAVID HAMPTON BOWEN.
LOUIS BRAUN.
CHARLES H. BUCHANAN.
FRANK W. BULLOCK.
ROBERT W. BUTLER.
WM. HARRIS COOK.
WM. HENRY CONIBEAR.
WM. H. DOOLITTLE.
JAMES DUNN.
FRANK W. EDWARDS.
JOSEPH H. ESDRIDGE.
FRANK B. FLORENTIN.
CYRUS W. FRANCE.
GEO. W. GAMMON.
JOHN R. GARDINER.
BYRON W. GRIFFIN.
ALLEN W. HAGENBUCH.
ROYAL G. HAMILTON.
JAMES M. HARMAN.
GUSTAVUS F. HARVEY.
JOHN HENRY HERON.
NOAH R. HOBBS.
SAMUEL J. HOLMES.
ROBERT HUTCHINSON.
JOHAN C. HVOSLEF.
OLIVER P. H. JEFFRIES.
FRANK SEBRA JONES.
HENRY W. JONES.
JOSEPH P. JOHNSON.
ALPHON. F. KALCKHOFF.
ANDREW KERSHAW.
ALFRED M. LANCASTER.
WM. M. LARRABEE.
FRANK LIGHTFOOT.
WM. M. MACFARLANE.
FINLA McCLURE.
JAMES D. McINTYRE.
JACOB MAY.
JAMES ALLEN MEADE.
JOHANN H. W. MEYER.
WM. WALKER MEYER.
EDW. WM. MINTON, A.B.
FRANCIS M. MOORE.
CHRIS. DEAN MOREY.
HIRAM IRVING NANCE.
FLOYD O'BRIEN.
MICHAEL T. O'CLERY.
SMITH ORR.
DAYTON PAINTER.
BRODIE W. PARKS.
CAMPBELL WM. PATRICK.
AUGUSTINE PERKINS.
HENRY PETTIBONE.
WILLIS F. PIERCE.
GEORGE F. PLEW.
GEO. W. RAMSEY, B.S.
WM. HENRY REEDY.
FRANK S. REYNOLDS.
LEONARD ROGERS.
CHARLES A. ROOD.
JOHN S. RYBURN.
CHAUNCEY M. SKINNER.
CALVIN K. SMITH.
EUGENE SMITH.
EUGENE R. SMITH.
THOMAS A. SMITH.
EDGAR SNYDER.
BENJAMIN E. STRICKLER.
AUGUST T. THIEMAN.
GEO. KING TILLOTSON.
CHARLES HENRY VENN.
CLARK RIENZI WARREN.
ROBERT R. WILLIAMS.
JOHN BRAND YOUNG.

AD EUNDEM.

JOHN A. STURGES, M.D.

HONORARY.

FOSTER PRATT, M.D.

Cause of the Vice-President's Death.—Dr. W. A. Hammond recently read a paper on the above-named subject before the New York Neurological Society. He thinks that death occurred from a lesion of the medulla oblongata. Well-marked calcareous degeneration of the basilar artery existed, and gastric ecchymotic spots and erosions of the gastric mucous membrane also existed, showing changes in the medulla oblongata. No clot of sufficient size to prove fatal, was found in the brain. Hence his conclusions.

---

The Appeal of the Committee of the Centennial Medical Congress at Philadelphia meets with cordial responses abroad. A medical Centennial Committee has been organized in Paris, and an office opened for the facilitating of its labors, at the Hôtel Cluny, Rue du Sommerard.

---

Professor Pitha, of Vienna, died December 28, 1875. In 1843 he was elected professor of surgery at the University of Prague; and in 1857 he was called to Vienna to fill the chair of surgery in the new military Joseph-Academy. The Vienna medical school has lost in Pitha an accomplished surgeon, an excellent diagnostician and a popular clinical teacher.

---

A Curiosity.—A deaf mute recently gave birth, in Paris, to her seventh child. She is married to a deaf mute. Five of her children were still-born and the sixth lives, talks and understands very well.—(*Journal des Sages-Femmes.*)

---

Small-Pox.—950 cases of this disease and 218 deaths, appeared in Cincinnati between January and November 1st, 1875.

DANCER'S CRAMP.—Dr. B. Schultz describes, in a recent number of the *Wiener Med. Wochenschr.*, a disease peculiar to solo dancers in the ballet, which has come under his observation in three cases, the most aggravated of which is described as follows: The patient complained of suffering very severe pains while dancing. Beginning in the soles of both feet the pains spread with increasing severity to the calves of the legs; they at last became so violent that her feeling of security was lost, the feet seeming as if made of wood. These pains were accompanied with violent palpitation; and, if she continued to dance, she felt faint and sometimes lost consciousness, the body becoming quite rigid. When the pain and palpitation were less intense, the pain continued after dancing, and ceased very gradually, leaving some tenderness of the soles; on attempting again to dance, the suffering would recur. Dr. Schultz found, from the examination of these cases, that the cause of the pain lay in the *pas* performed on the points of the feet, and is owing to exhaustion of the muscles which fix the metatarsus and phalanges of the great toe. The shoe worn by the dancer, without which the ballet step seems to be impossible, is made as follows: The dancing-shoe is made rather wide; the sole is of soft leather, and shorter than the foot, reaching only as far as the posterior third of the ungual phalanx of the great toe. The upper part, generally of satin, projects forward, and supplies the place of the deficient leather of the sole. This part of the satin is worked in threads, so that it may not be torn. In the interior of the shoe, over the leather sole, is a layer of thin, firmly pressed pasteboard, either extending over the whole breadth of the anterior part, or limited to the length of the great toe. In the former case it is carried back, gradually narrowed as far as the heel. The leather sole and its covering are lined with fine kid leather. The heel part of the shoe is quite soft, consisting only of satin; and the shoe is fastened above the ankle by narrow ribbons. Without this preparation the pointed step is impossible.—*N. Y. Med. Rec.*

---

A Night Service of the following character has just been established in Paris :

The physicians of each district are requested to state whether they will respond to calls made in the night.

The names and residences of those willing to respond to such calls, are inscribed in a record-book preserved in the police station of the district.

Those who have need of a physician in the night repair thither, and select any name they may desire from the list.

An officer, detailed for that purpose, accompanies the applicant to the physican's residence, escorts him to the patient's house, and, the visit over, reconducts him to his own home.

A small fee is charged for this service, payable by the family of the patient.—*Jour. de Méd. et de Chir.*

---

A new means of disinfecting the hands after making *post-mortem* operations, etc., is found, says the *Australian Med. Jour.*, in a combination of a drachm of etherial solution of peroxide of hydrogen and an ounce of Rimmel's toilet vinegar.

---

If a Piece of Nitrate of Silver should break off while you are cauterizing the throat, and it should be swallowed, the simplest way of preventing any cauterization of the stomach is to make your patient drink a large quantity of salt water. The silver will at once be turned to the insoluble chloride, and this will be vomited up.—*Memorabilien*, xx, 11.

Bergeron, in the Archives of Gynecology, calls attention to the difficulty of diagnosis, in a medico-legal point of view, in very young girls who have been indecently assaulted. It is well known that amenorrhœa may exist for several months in young and recently-married women, due solely to the first conjugal approaches. This is also often observed in young girls who have menstruated but once or twice. In seven cases the author has observed abdominal enlargement, with suppression of the menses, in young girls twelve, thirteen and fourteen years of age. He has also observed great abdominal enlargement, flattening of the costal regions, depression of the umbilicus, suppressed menstruation, enlargement of the breasts with brown areolæ of nipples, morning sickness, softening of the cervix, and a brownish line running from the mons veneris to the umbilicus, distinctly traceable. Pregnancy had not occurred. The medico-legal value of these observations is of some importance.

---

Dislocating the Jaw for Chloroform Narcosis.—Dr. Fleiberg, of Christiania, recommends the surgeon to deliberately dislocate the jaw, instead of pulling the tongue out with forceps, etc. It is easy to do this by placing both thumbs behind the symphysis, and both index fingers on the posterior edges of the rami, and dragging the bone forward. He seems to do it as a preventive, for he speaks of having done it *a thousand times.* Langenbeck tells us that he and Esmarch have often done this. Perhaps these surgeons will tell us if dislocation often occurs spontaneously to those who have thus been treated.—*The Doctor.*

---

During the Year 1875, twenty per cent. of all children born in the city of Mexico were illegitimate. The registered births amounted to 9,184.—*Anales de la Asociacion Larrey.*

